# Observing changes in human functioning during induced sleep deficiency and recovery periods

**DOI:** 10.1371/journal.pone.0255771

**Published:** 2021-09-01

**Authors:** Jeremi K. Ochab, Jerzy Szwed, Katarzyna Oleś, Anna Bereś, Dante R. Chialvo, Aleksandra Domagalik, Magdalena Fąfrowicz, Halszka Ogińska, Ewa Gudowska-Nowak, Tadeusz Marek, Maciej A. Nowak

**Affiliations:** 1 Institute of Theoretical Physics, Jagiellonian University, Kraków, Poland; 2 M. Kac Complex Systems Research Center, Jagiellonian University, Kraków, Poland; 3 Department of Cognitive Neuroscience and Neuroergonomics, Jagiellonian University, Kraków, Poland; 4 Małopolska Center of Biotechnology, Jagiellonian University, Kraków, Poland; 5 Center for Complex Systems & Brain Sciences (CEMSC3), Universidad Nacional de San Martín, Buenos Aires, Argentina; 6 Consejo Nacional de Investigaciones Científicas y Tecnológicas (CONICET), Buenos Aires, Argentina; Sapienza University of Rome: Universita degli Studi di Roma La Sapienza, ITALY

## Abstract

Prolonged periods of sleep restriction seem to be common in the contemporary world. Sleep loss causes perturbations of circadian rhythmicity and degradation of waking alertness as reflected in attention, cognitive efficiency and memory. Understanding whether and how the human brain recovers from chronic sleep loss is important not only from a scientific but also from a public health perspective. In this work we report on behavioral, motor, and neurophysiological correlates of sleep loss in healthy adults in an unprecedented study conducted in natural conditions and comprising 21 consecutive days divided into periods of 4 days of regular life (a baseline), 10 days of chronic partial sleep restriction (30% reduction relative to individual sleep need) and 7 days of recovery. Throughout the whole experiment we continuously measured the spontaneous locomotor activity by means of actigraphy with 1-minute resolution. On a daily basis the subjects were undergoing EEG measurements (64-electrodes with 500 Hz sampling frequency): resting state with eyes open and closed (8 minutes long each) followed by Stroop task lasting 22 minutes. Altogether we analyzed actigraphy (distributions of rest and activity durations), behavioral measures (reaction times and accuracy from Stroop task) and EEG (amplitudes, latencies and scalp maps of event-related potentials from Stroop task and power spectra from resting states). We observed unanimous deterioration in all the measures during sleep restriction. Further results indicate that a week of recovery subsequent to prolonged periods of sleep restriction is insufficient to recover fully. Only one measure (mean reaction time in Stroop task) reverted to baseline values, while the others did not.

## Introduction

Contemporary world is a sleep-deprived world. Apart from the professions ‘traditionally’ involved in so-called atypical work schedules (health services, entertainment, transportation, energetic and chemistry industries etc.) and suffering from sleep problems, there is a growing number of ‘regular’ day workers whose sleep-wake patterns become irregular due to periods of intense work requiring extra time and effort (infamous ‘deadlines’).

Those working from home, on the one hand enjoy the flexibility of their work schedules, more autonomy and adaptation of working times to individual needs, but—from the other hand—they observe blurring of the boundaries between work and private life, resulting in “living at work” and problems with time-management and self-discipline. The disruption of the rest-activity rhythm is one of the common side-effects of remote work.

Fortunately, the problems of young generations, suffering permanent social-lag due to their delayed sleep phase colliding with school timetables, are being noticed by scientists and authorities and first rearrangements of school starting times are introduced in some countries. Students are commonly subject to five-weekday sleep deprivation and weekend recovery (however, some ‘highly social’ individuals do not profit from rest, throwing themselves into intense and exhausting social life instead). Others, working on projects and coping with deadlines, experience even longer periods of chronic sleep restriction than just a week. Long weekend sleep possibly compensates for short weekday sleep in terms of mortality, as Åkerstedt et al. [1] showed in the analysis of a cohort of over 43 thousands of people during 13 years. How it works with prolonged sleep restriction and in other aspects than mortality is not yet fully understood.

Both partial (defined as a reduction in a sleep time over a 24-hour period, relative to individual sleep routine; also referred to as ‘sleep restriction’) and total (defined as a complete lack of sleep in a 24-hour period; also referred to as ‘acute’) sleep deprivation are linked with deficits in a cognitive performance [2], higher risks of motor accidents [3, 4] and medical errors [5]. Furthermore, insufficient sleep is also associated with ill health, such as a higher risk of diabetes, obesity, heart problems, and even stroke [6].

The impact of chronic sleep deficiency on human brain functioning is well documented (e.g. [7, 8]). Sleep deprivation impairs neurobehavioral functioning producing deficits in alertness, attention, memory, and executive functions (for a review, see: [9–11]) and affects locomotor activity [12, 13]. Current research results suggest the differences in the brain responses to acute deprivation and chronic sleep restriction as well as recovery processes [7, 14–16]. A limited number of studies on the recovery of neurobehavioral functioning after sleep deficit indicated a longer time for reversal of neural changes in the brain after chronic sleep restriction (e.g. [17–23]).

While the changes in the levels of neurocognitive performance (at least as it regards attentional processes) and sleepiness after acute total sleep deprivation may be interpreted in terms of homeostatic and circadian mechanisms of sleep-wake regulation, in case of chronic partial sleep restriction, this model seems to be incomplete. Hudson et al. [11] suggest the impact of the third process, the allostatic one, which refers to ‘sleep/wake history’ (a matter of days and weeks before) and may shift the setpoint of the homeostatic process. Sustained sleep restriction gradually shifts the homeostatic set point and consecutive days of recovery gradually shift it back.

Sleep deprivation, i.e. its consequences and recovery, may be considered on three levels: subjective, behavioral, and neuronal. The most often used subjective measures in sleep restriction studies are scales of self-reported sleepiness (e.g., Karolinska Sleepiness Scale, Stanford Sleepiness Scale, Epworth Sleepiness Scale, Accumulated Time Sleepiness Scale, Rotterdam Daytime Sleepiness Scale [24]. On the behavioral and neuronal levels the most widely studied cognitive domains are attention, working memory and executive functioning [25]. There is a long list of cognitive tests sensitive to performance deterioration during sleep deprivation (for a review, see: [14]. To assess behavioral effects of sleep deficit two measures of task performance are considered: speed (reaction times) and accuracy (for a review, see: [26]). Neural aspects’ analyses comprise fMRI (for a review, see [27] and EEG parameters: ERPs and power spectrum [28–30]. Finally, what is new in this field, changes in locomotor activity show to be promising indicators of sleep deficiency or of response to sleep deprivation treatment, what makes us think about actigraphy as a useful tool in sleep studies.

Even a seemingly mild reduction (of only a few hours) of sleep can have a significant impact on behavioural and neural functioning. Stojanoski and colleagues [31] have shown that participants (following only one night of restricted sleep equalling to five hours) presented decision-making processing difficulties and had reduced ERPs for motor preparation and execution, which had a detrimental impact on their vigilance. A recent study by Gibbings et al. [30] also found a decreased level of vigilance on behavioural measures after one night of sleep deprivation (5 hours) as well as intensified alpha-wave bursts that index the level of drowsiness. Additionally, they have found a reduced arousal as seen on the EEG power spectral analyses (such as increased frontal delta and occipital alpha, and reduced frontal beta waves).

Behavioural vigilance and EEG were also measured in a task involving driving performance following a night of normal and completely restricted sleep [32]. Significantly increased alpha and theta power spectra following a night of total sleep deprivation in frontal, central and parieto-occipital brain regions, compared to controls, have been found. Specifically, an increase in power was seen over the first 40 minutes of an hour-long driving task and was followed by a decrease in the last 20 minutes. An increase of beta power spectra was seen overall throughout the one-hour driving task and there were no differences between the normal and sleep-deprived groups. Increased alpha and theta power has been well documented to be associated with increased level of sleepiness and more fatigue (for example, [33–35]).

Understanding whether and how the human brain recovers from chronic sleep loss is important not only from a scientific but also from a public health perspective. The current study aimed at exploring, in natural conditions, the long term effects of chronic sleep deficiency on human functioning using behavioral, actigraphy, and EEG metrics. We evaluated the effects of 7 days sleep recovery following 10 days of sleep restriction on performance (accuracy and reaction times from Stroop task), spontaneous locomotor activity (distributions of rest and activity durations from actigraphy), and EEG parameters (amplitudes, latencies and scalp maps of event-related potentials from Stroop task and power spectra from resting states).

## Materials and methods

### Participants

The total number of 23 participants underwent our experiment. However, based on actigraphy recordings, 4 participants were removed from analysis due to failure to comply with the prescribed sleep restriction. The analyses of actigraphy take into account all the remaining 19 subjects (9 morning-oriented, 10 evening-oriented). Due to a revision of the Stroop task design, another 6 subjects are left out, with 13 subjects remaining in the reported EEG and behavioral analyses.

Further, individual days for a given subject were excluded as follows:

- if towards the end of baseline period a given subject slept as little as during sleep restriction period,
- if a given subject failed to comply with the sleep restriction (sleep more than 10% longer than prescribed) towards the end of its period, we removed its further days from the analysis, but left the recovery data,
- if in the middle of recovery a subject slept as little as during sleep restriction, we removed individual days from recovery data.

These exclusions result in 32 days (8, 11 and 13, respectively, for baseline, sleep restriction and recovery periods) out of 273 (13 subjects × 21 days). For actigraphy, 54 daily actigraphy recordings (14, 12 and 28) were excluded out of 399 (19 subjects), where some of the exclusions were additionally the first or last experimental day, which did not necessarily cover entire 24 hours.

The subjects were recruited based on Pittsburgh Sleep Quality Index [36] and Epworth Sleepiness Scale [37] questionnaires. All participants were healthy, drug-free (including alcohol and nicotine), and reported regular sleeping patterns with no sleep-related problems. The mean age of the 13 subjects was 21.5±1.3 y.o.; they were 12 women and 1 man; they had 6 evening-oriented and 7 morning-oriented chronotypes (defined by morningness-eveningness and subjective amplitude scales of the Chronotype Questionnaire [38]).

All of the subjects provided their written informed consent and were financially reimbursed for their time. The procedure was approved by the Jagiellonian University Bioethics Committee.

### Procedure

**Fig 1:**
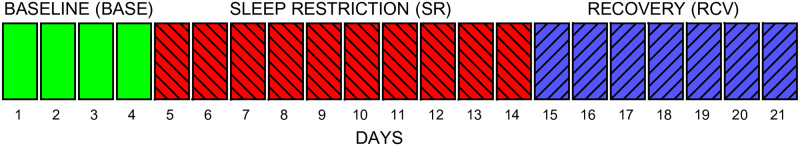
Study experimental design, showing number of days in each of the experimental sleep conditions.

As illustrated in Fig 1, the whole study lasted 21 consecutive days, which were divided into 3 sleep conditions: a 4-day period of unrestricted sleep (‘baseline’; hereafter abbreviated to BASE), then a 10-day period of daily 30% sleep reduction relative to individual sleep need (‘sleep restriction’; SR) followed by a 7-day period of unrestricted sleep again (‘recovery’; RCV).

The specific number of days in each phase was the compromise between researchers’ intentions to capture longer periods of sleep restriction and recovery than reported so far, and the comfort of participants which might have been jeopardized by too exhaustive procedure.

The subjects were instructed to conduct their normal daily routines during BASE; during SR they were individually prescribed a reduced sleep duration to follow; during RCV they were instructed to sleep without any restrictions. The subjects were additionally instructed to refrain from partying for the whole duration of the study and from caffeine consumption before visiting the EEG laboratory. Daily naps were not allowed in SR period. The subjects were informed that their sleeping patterns will be monitored with actigraphy. Data collected from participants who did not comply with the requirements were excluded from the study.

Individual sleep need was determined on the basis of two types of data: 1) the answer to the question “How many hours of sleep do you need to feel refreshed and active the next day?” (declared sleep need) and 2) sleep length calculated from actigraphy recordings during baseline. The SR protocol assumed 30% curtailment of this need. After excluding participants who did not manage to observe the rules of the study, mean baseline sleep was 7 h 37 minutes ± 50 min (SD over individual means) and during sleep restriction condition—5h 18 min ± 31 min (69.6% of baseline sleep). Average sleep length during recovery turned out to be again 7 h 36 min ± 26 min.

Daily, the severity of subjective sleepiness level was assessed by Karolinska Sleepiness Scale [39] and participant’s mood was assessed with the use of Positive and Negative Affect Schedule [40] questionnaire. Those data were reported in an article by Bereś et al. [41]. Mean sleepiness score during baseline was 3.4 ± 0.94, during sleep restriction period it amounted 5.55 ± 1.17, and in recovery it averaged 3.25 ± 1.09 pts.

### Data acquisition

**Fig 2:**
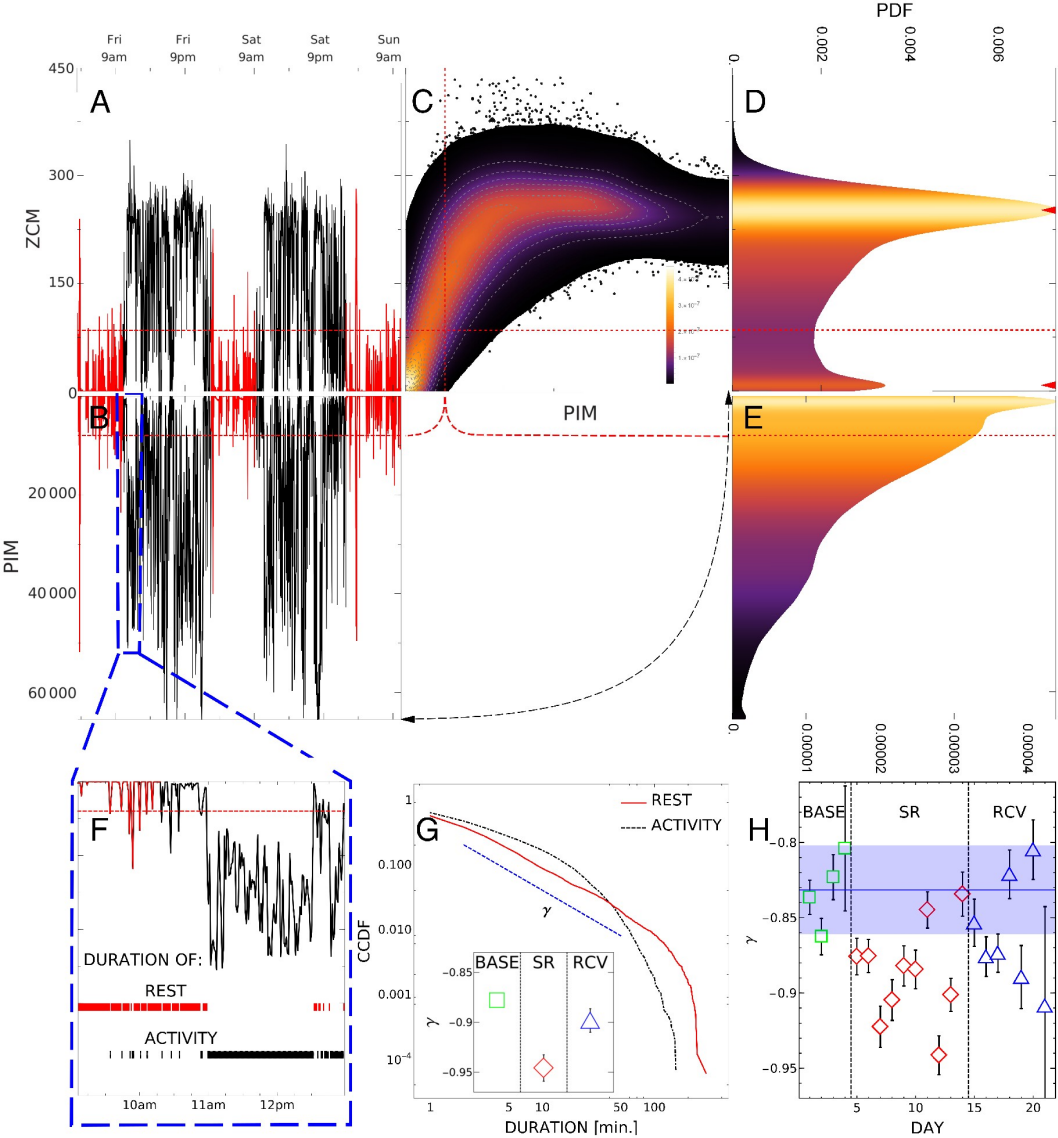
Summary of actigraphy analysis. A-B: a sample of the first two days (black) and three nights (red) of activity recording of a single subject in ZCM and PIM modes. C: density histogram of ZCM versus PIM activity (vertical and horizontal axes, respectively) collected from all subjects on all days (BASE, SR, and RCV) with the highest concentration of data around point (0, 0); the relation between ZCM and PIM is non-linear with ZCM saturating around 250. D-E: separate histograms of ZCM and PIM recordings, respectively; ZCM has two peaks, at 8 and 252, marked by red triangles corresponding to diurnal and nocturnal activity, while PIM is unimodal. F: rest and activity periods (red and black stripes) are defined as segments of activity continuously smaller or greater than a threshold (red dashed line); lengths of these segments are collected into duration distributions in G. G: complementary cumulative distributions of rest and activity durations in SR period of all subjects in log-log scale; *γ* is the exponent of power-law tail of rest CCDF (slope of the blue dashed line). Inset: *γ* exponents in baseline, sleep restriction and recovery conditions. H: *γ* exponents for each day of the experiment obtained from rest duration CCDF of all subjects.

The motor activity of participants was continuously recorded with actigraphy (Micro Motionlogger Sleep Watch, Ambulatory Monitoring, Inc., Ardsley, NY) in order to control their sleep timing and duration; a given participant wore the same device during the whole 21-day period. The actigraphs collected data in 1-min intervals in two available modes: the zero-crossing mode (ZCM; i.e., frequency of activity signal crossing a zero threshold) and the proportional integrating measure (PIM; i.e., intensity or under the signal curve), see Fig 2A–2E. The EEG recordings were performed in the laboratory, whereas the actigraphy data were collected continuously both in the laboratory and in the natural environment.

**Fig 3:**
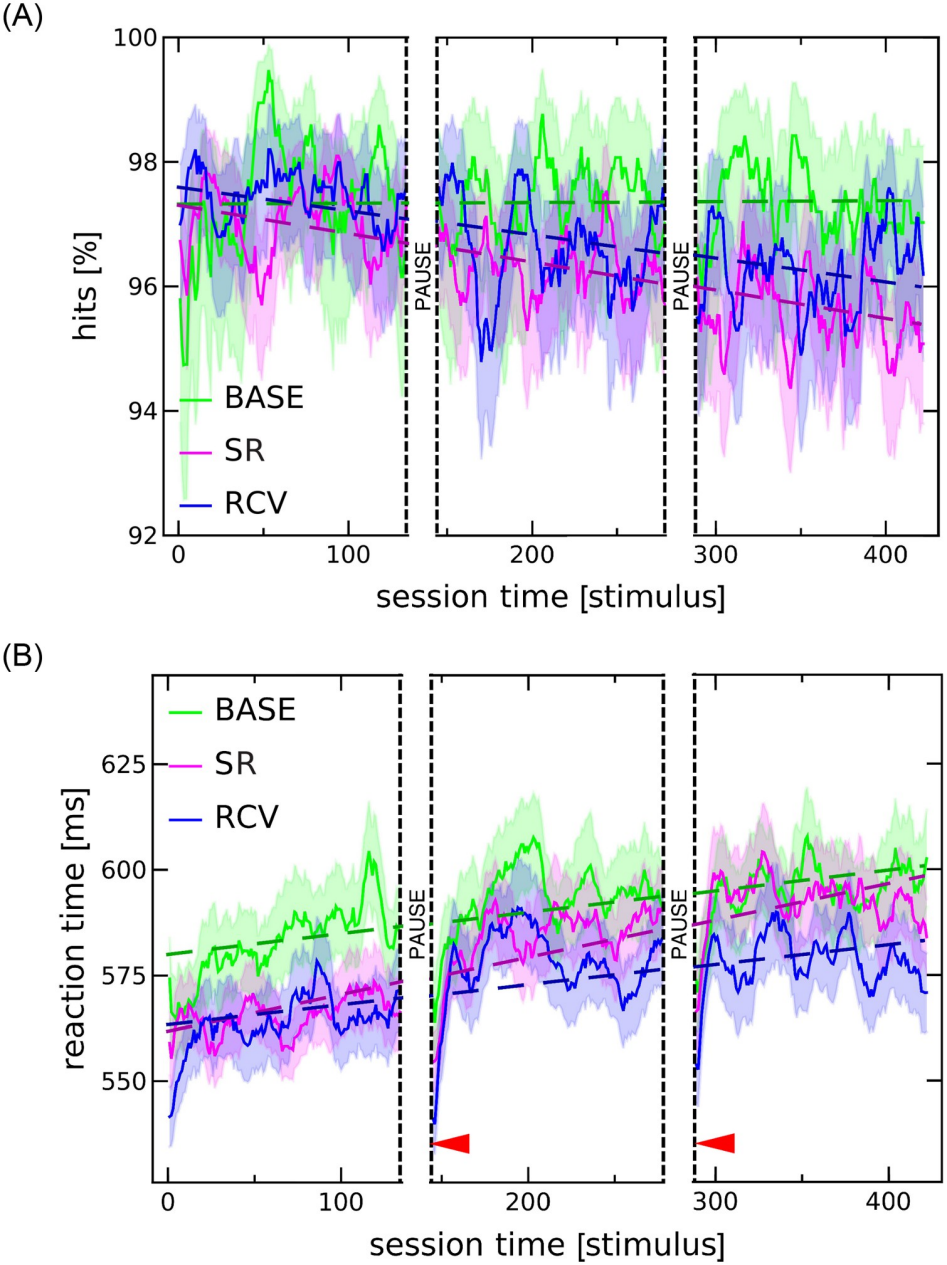
Accuracy (top) and reaction times (bottom) as functions of time spent on performing the task (measured by the number of stimuli presented). Vertical dashed lines indicate pauses between blocks of the Stroop task. The curves represent means over subjects and days within a given condition. and the shaded regions 95% confidence intervals; The straight lines are linear fits; the RT slope is significantly higher in SR. Red triangles point to RTs dropping just after the pause, which happens in all conditions.

Each day the subjects’ performance in terms of cognitive information processing was measured in a classic Stroop test. The subjects were asked to decide whether the name of the color matches with the ink in which it is written (congruent conditions) or not (incongruent conditions). So the more automated task (reading the word) interfered with the less automated task (naming the ink color) resulting the difficulty in inhibiting more automated process known as the Stroop effect. In total there were *N*_*s*_ = 432 randomly shuffled stimuli presented in 3 separate blocks (144 stimuli each) with a short break in between each block, as indicated in Fig 3. Half of the stimuli were congruent, and half were incongruent. The inter-stimulus interval between each stimulus was between 1500 ms and 3500 ms (in steps of 400 ms, with 2500 ms on average); the entire task lasted 22 minutes and 11 seconds on average.

All participants underwent one training session before beginning of the experiment in order to avoid the learning effect.

Each day, participants’ brain activity was being monitored with an EEG (64 electrodes, 500 Hz sampling frequency; Geodesic Sensor Net, EGI System 300, USA), while they were in a resting state (for 8 minutes with open eyes, RSeO, followed by 8 minutes with closed eyes, RSeC), and next performing Stroop test. The EEG session for a given participant took place in the laboratory at the same time every day, in the morning (8–11 a.m.) or evening (6–9 p.m.) according to the participant’s diurnal preferences.

### Data analysis

Whenever possible, when reporting on statistical significance of a result we provide (in the text or in the S1 File) a bare p-value. Unless otherwise explicitly stated we use the term “significant/insignificant” for significance level *α* = 0.05 with multiple-comparisons taken into account (Holm-Bonferroni method with three comparisons: BASE-SR, BASE-RCV, and SR-RCV). Particular methods and tests depend on data type, and are described in detail below.

#### Actigraphy

The ZCM and PIM modes were the only modes of data acquisition available for the devices used. We decided to record and analyze both of them for both theoretical and practical reasons: a) on one hand ZCM has been the most popular choice in the literature, while on the other PIM has a higher resolution, b) the two modes measure different physical quantities (in a nutshell, the frequency or intensity of movements, respectively for ZCM and PIM) which offer complementary views on the nature of recorded movements, c) *a priori* both quantities (number of activities and the energy expended on them) could have been relevant for sleep deficit, d) as evidenced by panel C in Fig 2, the two modes are non-linearly correlated and so they do carry different information (in particular above ZCM value 200), e) as shown in Fig 9 in S1 File, the values of relevant exponents derived from both modes differ, hence we keep both for better replicability.

We followed the procedures presented in the detailed actigraphy analysis of sleep deprived individuals in [12, 42] (on a different data set). First, the raw actigraph data *X*(*t*), see Fig 2 panels A and B, were segmented into 18-hour days (4 hours sleep followed by 14 hours wakefulness); data which did not fit into these durations or contained other artifacts (5.2% of all data) was discarded. The fixed length removes statistical artifacts connected with inter- and intraindividual variance of sleep duration and the forced difference between restricted and unrestricted sleep. Next, based on a predetermined threshold, the data was split into ‘activity’ and ‘rest’ (data points above and below the threshold, respectively), see Fig 2 panel F. Lastly, their durations (lengths of uninterrupted periods of activity or rest) were extracted. The thresholds were set to *T*_*ZCM*_ = 85 and *T*_*PIM*_ = 8000, based on the optimal goodness of fit of a power law (3) to the distribution of rest durations.

Since the ZCM and PIM actigraphy modes may contain complementary information, in the present paper we added a new methodological element analyzing four scenarios:

- threshold only for ZCM, see panel A in Fig 2,
- threshold only for PIM, see panel B,
- jointly: when *both* ZCM *and* PIM cross a threshold, see panel C,
- jointly: when *either* ZCM *or* PIM crosses a threshold, see panel C.

For a given scenario, we counted the number of activity/rest periods of given duration time jointly for all subjects, and calculated the resulting probability density function (PDF) *p*(*τ*) of duration time *τ*. To better assess statistics of rare events in tails of PDF we construct, as the main measure of discussed phenomena, the complementary cumulative distributions (CCDF) *C*(*a*) of durations *a*, see Fig 2G:

$$\begin{array}{c} C\left(a\right)=1-\int_{-\infty }^{a}p\left(\tau \right)d\tau =\text{Pr}\left(\tau \geq a\right)\equiv \int_{a}^{\infty }p\left(\tau \right)d\tau , \end{array}$$ (1)

which represents surviving probability for the system to stay in a given state for up to the time *a*.

The function *C*(*a*) is equal to one for a minimal value *a* = 0 and tends to zero for *a* → ∞. For a stationary time series the survival probability *C*(*a*) is expected to have a characteristic scale (relaxation time *τ*_*rel*_) related to the probability per unit of time λ to undergo the change of the state. The rate λ, named the hazard rate in the theory of critical phenomena, denotes then the ratio

$$\begin{array}{c} \lambda =\frac{\text{Pr}(\tau <a\leq \tau +d\tau )}{C(\tau )d\tau } \end{array}$$ (2)

and can be associated with *C*(*a*) representing a simple exponential function of dwell times, *C*(*a*) = *e*^−*λa*^ with λ = 1/*τ*_*rel*_. As discussed elsewhere [12, 42, 43], in case of disordered systems, the notion of the survival function and the corresponding (hazard) rate λ(*τ*) can present much more complex behavior, strongly deviating from a simple Poissonian-like occurrence of the ‘threshold-exceeding’ events.

For the purpose of statistical analysis, the numerical estimates of cumulative distributions was fitted with two mathematical formulae, power-law of the form

$$\begin{array}{c} C\left(a\right)∼a^{-\gamma } \end{array}$$ (3)

for rest periods and a stretched exponential

$$\begin{array}{c} C\left(a\right)∼\exp(-\alpha a^{\beta }) \end{array}$$ (4)

for activity periods.

For robustness, the cumulative distributions were bootstrapped (on the BASE/SR/RCV period level there were 1000 samples with 60 daily recordings each; on the day-by-day level there were all combinations of *N*_*d*_ − 2 daily recordings, ranging 10–171 samples a day, where *N*_*d*_ is the number of available recordings for a given day). The ordinary least-squares fitting was performed on log-log or log-linear data, respectively for a power-law and stretched exponential, in order to account for the tails in the distributions. The fitting was weighted, with weights coming from bootstrap standard deviations, which removed variance caused by the very end of the distribution tails. The fitted parameters *α*, *β* and *γ* were then compared for several period and subject combinations. The error bars in Fig 2G and 2H are 95% confidence intervals of the respective fit. The statistical significance of differences between exponents *γ* was tested by t-test [44, 45] for differences in linear regression slopes (version with unequal residual variances; the test is based on fit residuals, which in this case are very highly non-normal due to the very end of the distribution tail, cf. rest distribution in Fig 2G; to fulfil normality assumption we took approximately only the first half of residuals).

#### Behavioral measures

The two quantities scrutinized in this paper are: accuracy (percentage of correct responses) and reaction times, for results see Sec. Stroop test—time-on-task effect. We report their change over the duration of an experimental session; for reaction times, we also report their decrease shortly after pauses dividing the session into blocks. As a proxy of time passed in a session, we count the number of stimuli shown.

The curves shown in Fig 3 are averages over subjects and days within a given condition (baseline, sleep restriction, recovery). For visual clarity, the curves are moving averages over *w* = 10 consecutive stimuli; moving average was not used for the analysis. Since the responses have binary values (correct or error) and the percentage of hits is high (around 96%), the accuracy has a highly skewed binomial distribution. Consequently, to draw error bars in the figure, we use Clopper–Pearson binomial proportion 95% confidence intervals. For reaction times, we use standard 95% CI.

The dashed lines in Fig 3 represent fitted linear models. The linear regression did not take into account the pauses in the experiment (nor the first 6 stimuli in each block for RTs), i.e., the input data were only the 432 (414) stimuli responses.

The change in accuracy and reaction times reported in Table 2 in S1 File is simply the slope of the regression line multiplied by *N*_*s*_ − 1.

The 95% CI given in the table are computed from the standard error of the fit. The statistical significance of differences between the slopes can be assessed simply by investigating the confidence intervals, but we also provide p-values from the appropriate t-test [44, 45] (version with unequal residual variances). One must bear in mind, however, that residuals of accuracy and reaction time do not fulfil normality assumption. Since at each time point the distributions of inter-individual and inter-day reaction times is skewed, see Fig 10 and Table 2 in S1 File we also report results on linear fits to time-point medians of RTs, in which case the fit residuals are normal.

The effect of after-pause speed-up was measured by RT residuals from the linear fit described above. RTs to the first 6 stimuli in the 2nd and 3rd block of each experimental session were used. We used RTs from all valid subjects and days within a given condition (BASE, SR, RCV). Since their distributions are non-normal and the variances are not equal, instead of ANOVA we used Kruskal-Wallis test [46] followed by Conover-Iman post hoc [47] (chosen instead of the usual Dunn’s test for its reportedly greater power) as implemented in R [48] to test for stochastic dominance of the samples.

#### EEG

The EEG analyses were performed with the use of EEGlab v. 13 [49] and ERPLAB 6.0 [50] on MATLAB R2016b. The time series sampled at 500 Hz were digitally filtered (0.5–35 Hz), the reference was recomputed from Cz electrode to average, bad channels and time epochs with artefacts were removed. An Independent Component Analysis (ICA) was used to separate and remove physiological artefacts, including saccade-related spike potentials [49]. For recording and all analyses the EGI’s 64-channel Geodesic Sensor Net system was used. We report in detail on electrodes *vref*, 4, 34, 35, 39, 12 and 60. The equivalents of these electrodes in 10–10 system (within 1 cm accuracy) are Cz, Pz, FCz, O1, O2, F3 and F4, respectively. Henceforth, we use the 10–10 names for better readibility. In total there were 32 recordings for baseline, 47 for recovery and 69 for sleep restriction.

#### EEG: ERP analysis

The event-related potentials are reported in detail at FCz and Pz. The electrodes were chosen by the maximal absolute difference between baseline activity and either SR or RCV period activity: for BASE-SR it was electrode Pz and for BASE-RCV it was electrode FCz. The times (166, 268, 438, 600 ms) at which topographical maps are plotted were also chosen as the times when the maximal differences at either FCz or Pz occurred.

**Fig 4:**
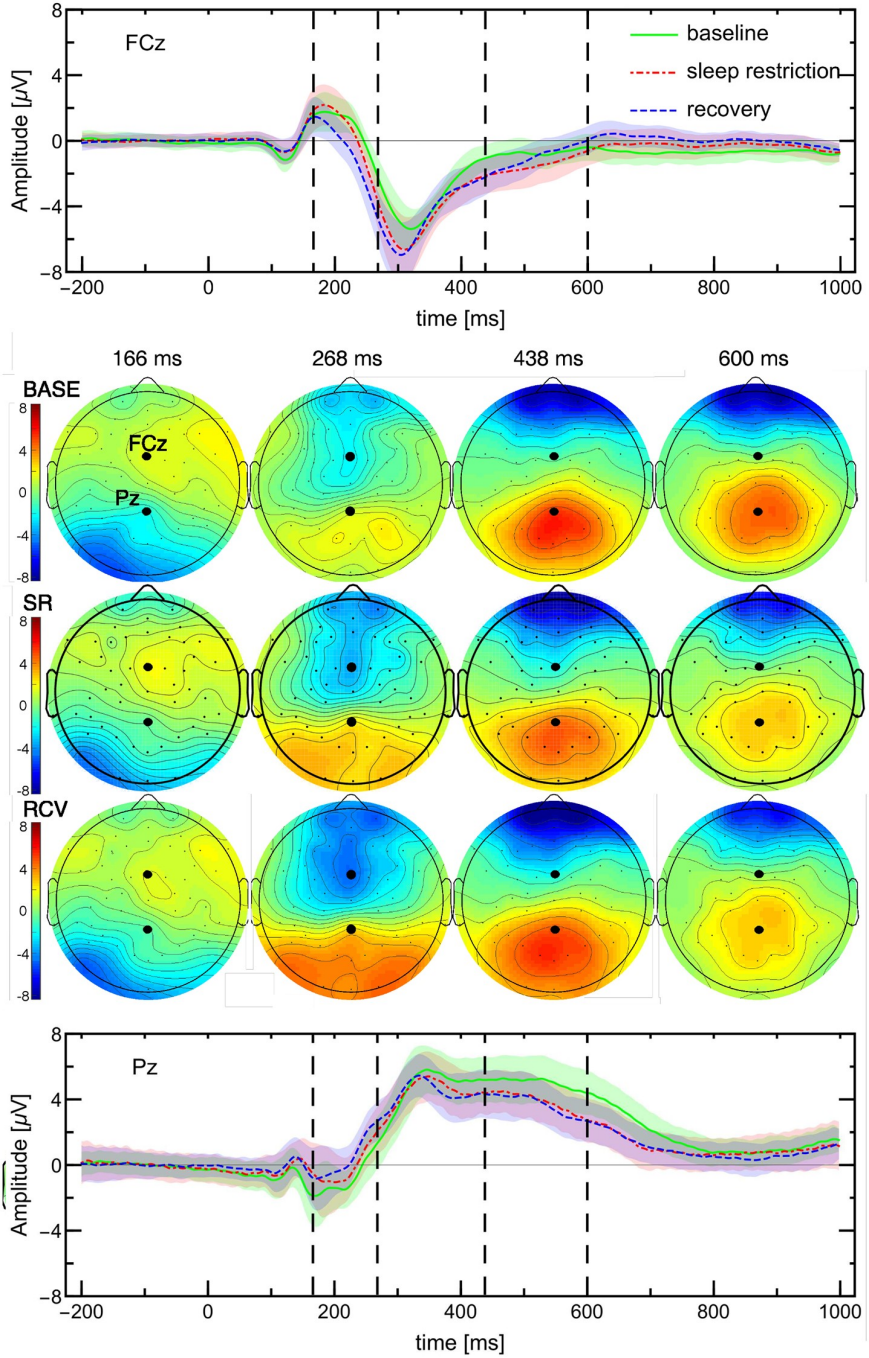
Activation maps and ERPs in response to Stroop task (average of congruent and incongruent stimuli) with 200 ms baseline. The ERPs of BASE, SR (days 10–14), and RCV are shown at the two locations (FCz and Pz) that correspond to the largest differences between these conditions, cf Fig 5. Four time points (dashed vertical lines at 166, 268, 438 and 600 ms) are chosen on the same basis. The shaded areas are standard errors (mean SE ≃1.1 *μV*). The maps are shown in columns corresponding to these times points; their amplitude scale is the same as in the ERP plots. The differences visible in maps at 268 ms can be attributed to change in latency.

**Fig 5:**
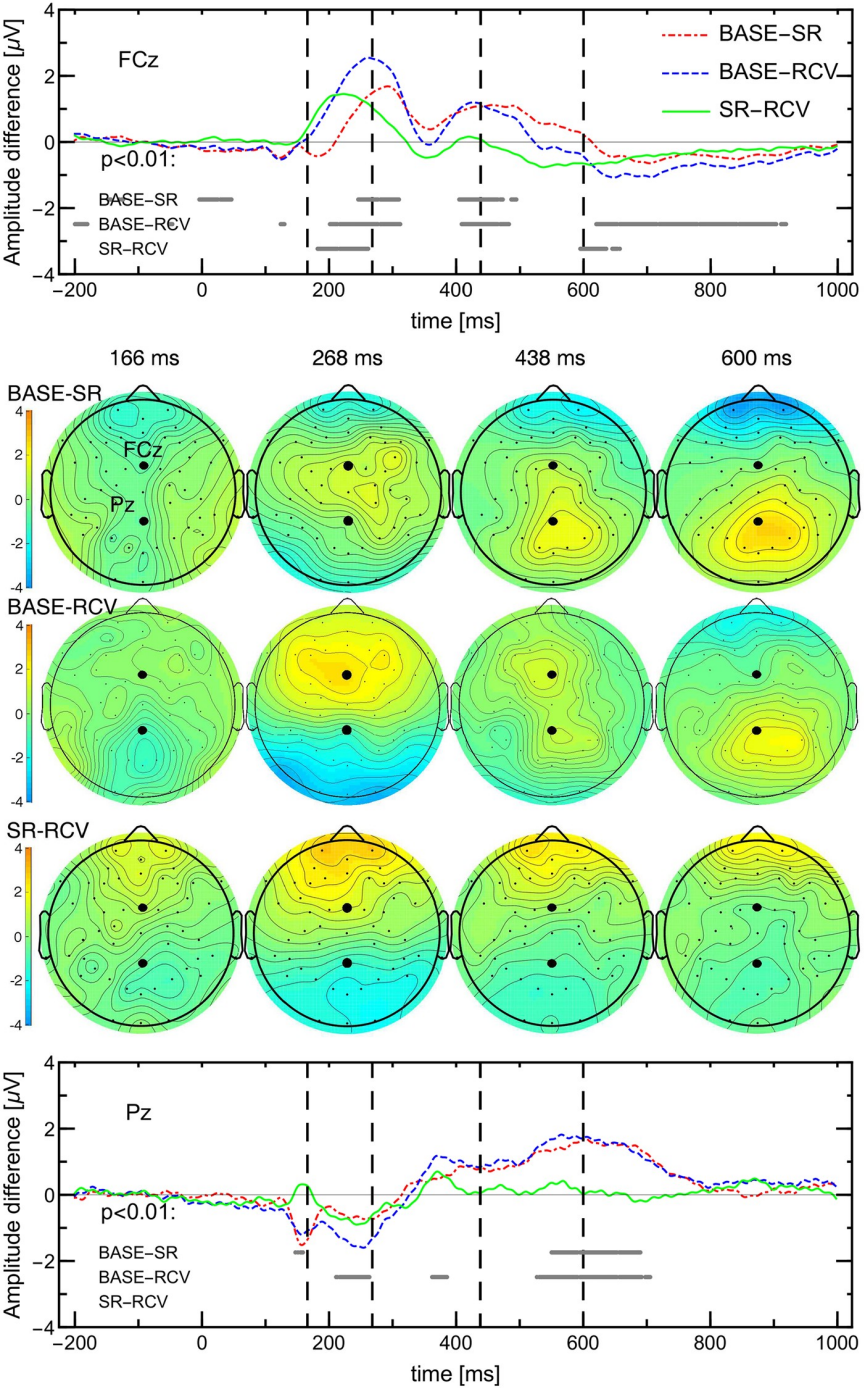
Differences in ERP amplitudes between BASE, SR (days 10–14), and RCV periods, derived from Fig 4 (note that the scale is twice as small as therein). Grey lines in the lower part indicate time points at which t-tests for pairwise comparisons yielded p-value *p* < 0.01. SR and RCV only differ frontally in early processing, whereas they diverge from BASE in frontocentral and parietal areas for extended intervals. The differences around 268 ms can be attributed to latency change.

The ERPs were measured with segments from 200 ms before to 1000 ms after the onset of the target stimulus, with the [-200 ms, 0 ms] pre-stimulus baseline mean subtracted. The potential amplitudes are grand averages, i.e., averages over ERP waveforms from all daily sessions of all subjects in a single condition (BASE/SR/RCV) and stimuli type (congruent/incongruent). Since, as reported in in [41], there was no significant interaction between the sleep condition and stimuli type, subsequently the ERPs from congruent and incongruent stimuli were averaged for robustness and a more concise presentation in Figs 4 and 5.

Presented measurement uncertainties are pointwise standard errors of the mean as implemented in ERPLAB. The significance tests between condition samples, as presented in Fig 5, were performed at each time point separately with a t-test or Mann-Whitney test, depending on which test assumptions were met. In the figures referenced above we show only the late SR period.

Latency shifts between conditions were computed with ERPLAB [50] as onset latency at 50% peak height.

#### EEG: Power spectrum analysis

After the pre-processing described in Sec. Data analysis: EEG above, the power spectra of continuous EEG signals were computed in resting state with eyes open and closed for chosen electrodes using Welch’s overlapped segment averaging estimator. Powers were then computed separately for each subject on each day in four sub-bands: delta (1–4 Hz), theta (4–8 Hz), alpha (8–13 Hz), and beta (13–30 Hz). Statistical tests were performed in each band separately, between distributions of measurements collected from all days and subjects in a given sleep condition (BASE/SR/RCV). The distributions were in general non-normal (the highest p-value 0.066 in Shapiro-Wilk test was obtained for beta band in baseline period; others were orders of magnitude smaller). Consequently, we used Kruskal-Wallis test followed by Conover-Iman post hoc to test for stochastic dominance of the samples.

We also examined how brain waves changed in time during the resting state part of the experiment (both eyes open and closed), since the power estimates of the EEG activity were reported to depend on the length of the recording and the analyzed segments, and the way they are selected [51]. We specifically focused on alpha rhythms, due to their known correlation with sleepiness [33], considerable inter- and intra-individual variability [52, 53] and time-dependent variability [51, 54]. The powers were computed jointly for channels O1 and O2, divided into 5 time blocks, 96 *s* each.

## Results

### Actigraphy

Fig 2 summarizes actigraphy data characteristics and processing. A sample of raw ZCM and PIM time series is shown in panels A-B. As visible in panel C, the dependence between the two modes is linear only for small values. The resulting distributions of activity as recorded in ZCM and PIM (panels D-E, flipped to the side) also differ: ZCM has two clear peaks corresponding to daily activity and sleep, while PIM distribution monotonically falls down from low to high intensities. Panel F shows periods of activity and rest for PIM (respectively above and below *T*_*PIM*_ = 8000) obtained from an exemplary recording.

First, the activity period durations undergo stretched exponential distributions as found in our previous papers [12, 42]. In this experiment we found some dependence of the exponent *α* on the measurement period, contrary to our previous results. This may be due to different experimental conditions (21 consecutive days as contrasted to two separate 7 days periods). The exponent *α* in 4 different mode combinations can be found in Fig 9 in S1 File.

More importantly, we concentrate on the CCDF of resting period durations *C*(*a*). In Fig 2G and 2H, we present the *γ* exponents (in the PIM mode at the group level) as a function of the measuring period and as a function of consecutive days. Consistently with our earlier work [12, 42] the distribution’s slope changes rapidly once the sleep loss appears (statistically significant with *p* = 0.0017).

The new result is long recovery time: even one week of regular sleep does not stabilize the distributions (the difference between SR/RCV is significant, *p* = 0.019, but smaller, while for BASE/RCV it is still visible but below confidence threshold, *p* = 0.16). Similar effect, although exhibiting more variability than in the PIM mode, is observed in the ZCM mode, cf. Fig 9 in S1 File.

Having at our disposal two different measuring modes we posed the question which mode or combination of modes is more informative of the changes resulting from sleep loss. The question follows from the observation that both modes are correlated but the relation is not linear, Fig 2C. Consequently, the division into resting and activity periods can differ in both modes. Thus, as indicated in Sec. Data Analysis: Actigraphy, we additionally repeated the tests reported above for exponents of distributions generated by the two modes simultaneously: either by defining resting states by alternative of ZCM and PIM thresholds (below ZCM or below PIM) or their conjunction (below ZCM and below PIM). Comparison of the two measurement modes and their combinations is summarised in Fig 9 in S1 File. The left panel of that figure shows that the use of both modes does not enhance the *γ* exponent result and the PIM measurement mode seems to be most suitable for this particular experiment.

All the above combinations show a general trend—a significant change of the exponent during sleep restriction period and a partial return towards baseline values.

### Stroop test—Time-on-task effect

The sleep restriction period influenced participants cognitive functioning as tested with the Stroop task. The main observation is poorer behavioral performance on the Stroop test during sleep restriction period followed by a gradual, incomplete recovery, as reported in [41].

Our previous report also showed that there was a tendency to have fewer correct responses during sleep restriction period -– mainly due to a significantly higher rate of errors and omissions, and a slightly increased rate of double responses. A decreasing tendency in reaction times (RT) for the first few days of the experiment was also observed, which can be interpreted as learning the Stroop task. As expected [55], the distributions of RTs for congruent stimuli have smaller means and variances than for incongruent ones. Somewhat counter-intuitively, for incongruent stimuli there are more correct responses in the entire experiment than for congruent (see section “Behavioural results” in [41]).

Here, we focus on how the time spent on performing Stroop task (within a given experimental session) affects accuracy and reaction times, see Fig 3. The technical details on statistical computations are given in Sec. Behavioral measures.

The accuracy of responses, systematically and significantly decreases during session for SR and RCV conditions (by 1.96% and 1.64%, respectively), but not for the baseline period. The difference in the slopes between BASE and either SR or RCV is highly significant (*p* < 0.001; see Table 2 in S1 File for confidence intervals and p-values). In other words, subjects undergoing and recovering from sleep restriction found it harder to maintain perfect accuracy during the 20-minute experimental session.

The change is more pronounced for reaction times. During a given session the RTs slightly increased, as visible in Fig 3 (bottom). The difference between the last and first stimulus in a session, as given by linear regression, is 24.3 ms, 37.5 ms, 21.6 ms for BASE, SR and RCV conditions, respectively. As already visible, the difference between BASE and RCV is not significant, but it is for BASE/SR and for RCV/SR (*p* < 0.001; see Table 2 in S1 File for details).

It should be noted, on one hand, that for accuracy and mean RTs the normality assumption on regression residuals is not fulfilled, and so the reported p-values for slope differences should be treated with caution. On the other hand, the confidence intervals obtained from the fitting do corroborate the conclusions. Additionally, due to high skewness of reaction time distributions [55] (see Fig 10 in S1 File), means might not be appropriate estimators, hence in Table 2 in S1 File we also include results for median (where both differences for BASE/SR and RCV/SR are below *α* = 0.05 significance level, but marginally do not survive Holm-Bonferroni correction). We nevertheless believe that mean RTs—thanks to their sensitivity to skewness and outliers—can actually carry more information on atypical behaviour induced by sleep loss than medians.

In the Fig 3, there are two dips in the RT curve: in one-third and two-thirds of the session, which is due to pauses between blocks of Stroop task (during which the electrodes were watered, and consequently the subjects were able to rest). These after-pause dips basically reset the reaction time to what it was at the beginning of the session. They take 6–16 consecutive stimuli, and are on average 20.8, 21.8, and 37.6 ms deep for BASE, SR, and RCV, respectively. Kruskal-Wallis test indicates differences (*p* = 2.5 × 10^−5^). Post-hoc test shows that the difference between BASE and SR or RCV is significant, but is not for SR and RCV, see Table 2 in S1 File.

In summary, the ratios of accurate responses and omissions directly follow the schedule of sleep restriction; they go back almost to normal already after the first day of recovery period. The difference between conditions in linear growth of reaction times (and linear decrease of accuracy) indicates that the more sleep deprived subjects get tired more quickly. A difference between conditions in the after-pause drop in reaction times indicates that the pause gives more rest to the sleep deprived and recovering subjects. An interesting general observation is that the variability of response accuracy does not average out as well as the variability of reaction times.

### EEG data: ERP analysis—Scalp maps and electrode dependence

The effects related to task performance in different conditions can be observed in event-related potentials.

It was observed that the P300 neural response was attenuated during and after sleep loss as compared to the baseline [41]. Here, we analyse the whole scalp maps of ERP amplitudes to discover how the brain response changes during different sleep condition.

The ERPs having the maximal absolute difference from BASE period are located at Pz for SR and FCz for RCV, as shown in Figs 4 and 5. Dashed lines in the ERP plot indicate the times (166, 268, 438, 600 ms) when the maximal differences occurred. Consecutive columns of maps correspond to these time points.

The general observation from Fig 4 is that post-stimulus activations are mostly bipolar with strong positive activations in occipital and centro-parietal regions. Fig 5 additionally shows where and when the differences occur: visibly the baseline activations are greater at first in the frontal, then central, and finally parietal regions. Also visibly subjects in SR and RCV periods had stronger occipital activations earlier and the parietal activations disappeared sooner. Note that the greatest differences in ERP curves occur around and not at their peaks, which rather suggests a delay between them. The BASE onset latency at FCz is 272 ms and at Pz 292 ms, as visible in Fig 4. The time shift from the baseline ERP curve for FCz and Pz is: −8 ms and −10 ms, respectively, for SR and −16 ms and −24 ms for RCV.

### EEG data: Power spectrum analysis

The shapes and variances of the band-power distributions differed between groups. The Kruskal-Wallis tests for differences between BASE, SR, and RCV periods yielded statistically significant results only for resting-state condition at central and occipital electrodes, see Tables 3 and 4 in S1 File. The differences were discovered between **BASE** and **RCV** in **delta**, **theta** and **beta bands** at Cz in RSeO and between **BASE** and **SR** in **delta** in RSeO and **alpha** in RSeC at O1 and **alpha** in RSeC at O2.

**Fig 6:**
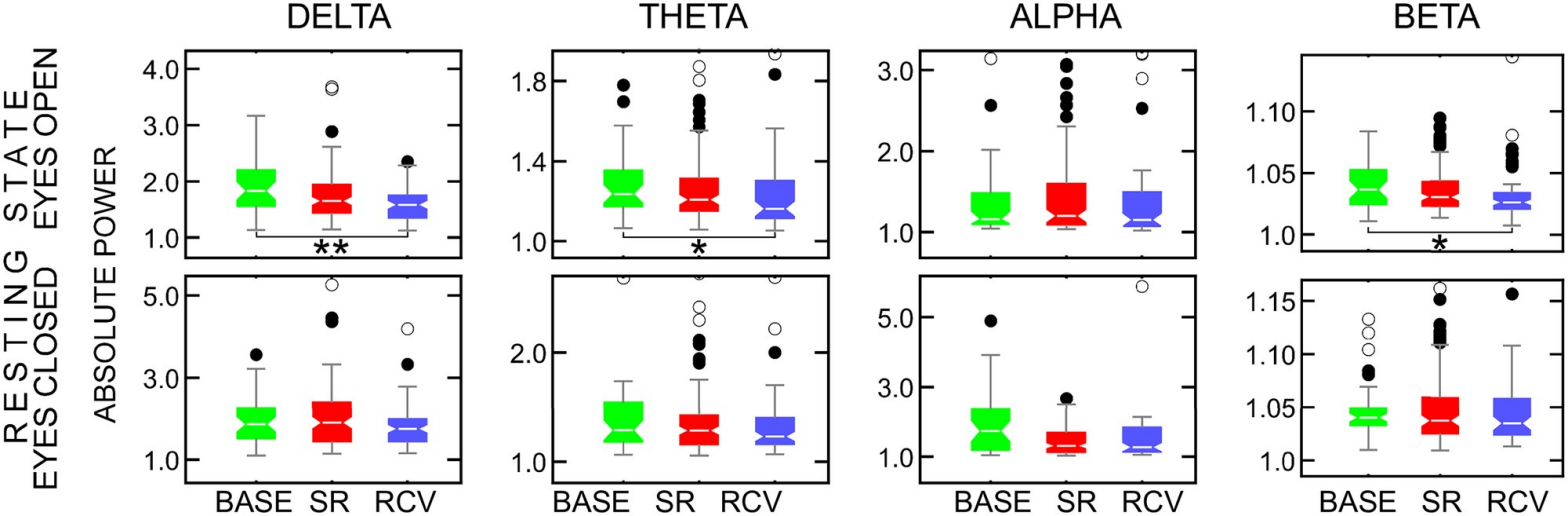
The absolute power of *δ* (1–4 Hz) (first column), *θ* (4–8 Hz) (second column), *α* (8–13 Hz) (third column) and *β* waves (13–30 Hz) (fourth column) for resting state with eyes open (top row) and closed (bottom row) at Cz electrode. The boxes show median and interquartile range, together with near (full circles) and far (empty circles) outliers. Some extreme outliers are not shown to retain the scale for visibility. Significant differences between conditions are marked with: * *p* < 0.05, ** *p* < 0.01.

On closer look, the differences at Cz came from a weak negative correlation with time, i.e., gradual decrease of power in these bands as the experiment progresses from BASE phase, to SR, and to RCV, which is visible in the top row in Fig 6. The behavior of alpha band in resting state condition with eyes closed also did indicate differences between the periods, which were however below statistical significance. The highest power of the *δ* and *θ* oscillations at Cz were observed in the Stroop task. In contrast, the power of *β* waves during the task was drastically low. Nevertheless, there were no significant differences between conditions in the Stroop task.

**Fig 7:**
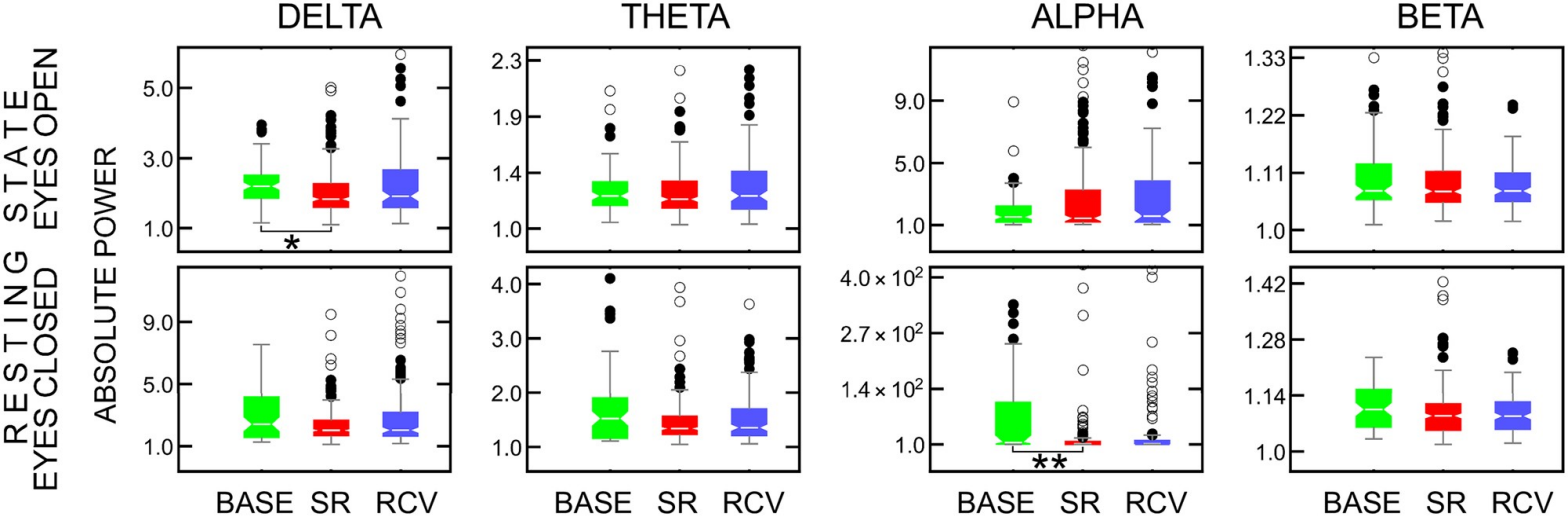
The absolute power of *δ*, *θ*, *α*, and *β* waves for resting state with eyes open (top row) and closed (bottom row), as in Fig 6, jointly from channels O1 and O2. The p-values for differences at each channel can be found in Table 3 in S1 File.

As expected, the *α* rhythms at occipital electrodes were significantly higher when subjects had their eyes closed when compared to performing the task or resting state with eyes open. The results at O1 and O2 are consistent, with alpha and delta power greater in BASE period than in SR, see Fig 7 and Tables 3 and 4 in S1 File. The frontal electrodes F3 and F4 show no statistically significant differences, although the tendency in alpha band in resting state with eyes closed is similar to that of O1 and O2.

**Fig 8:**
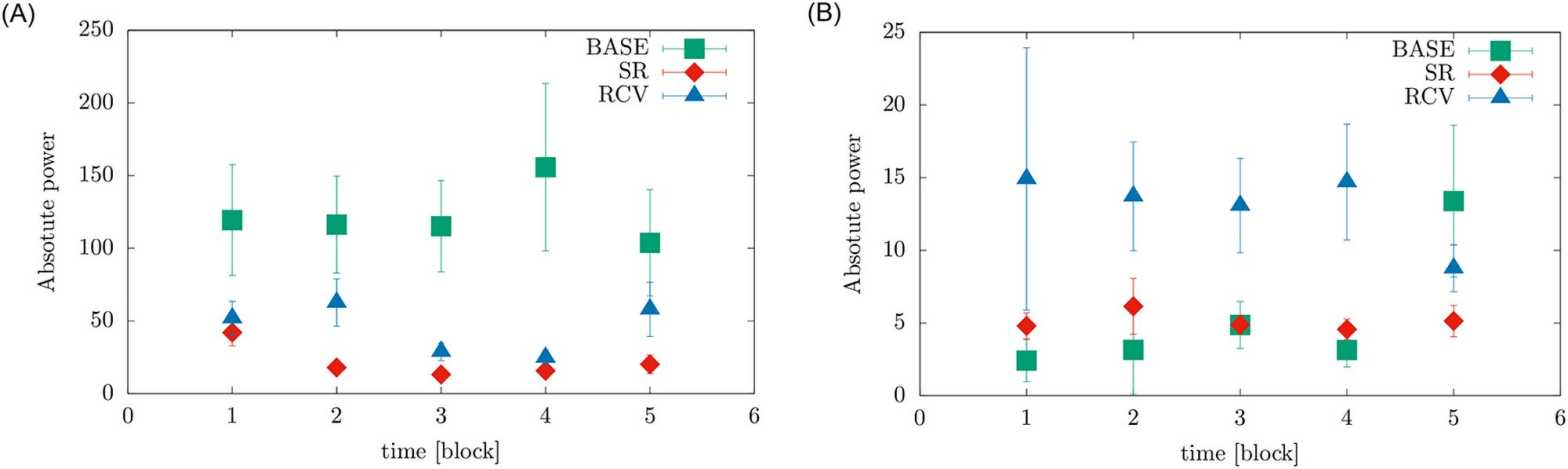
The power spectrum of alpha waves changing in time for resting state with eyes closed (left panel) and eyes open (right panel). Data from electrodes O1 and O2.

We also examined how brain waves, in particular alpha waves, changed in time during the resting state part of the experiment. Fig 8 illustrates the alpha rhythms in both resting states (eyes open and closed), jointly for channels O1 and O2, divided into 5 time blocks, 96 *s* each. This plot clearly presents two effects: firstly, the lowest power for the sleep restriction period; and secondly, decreasing the value of the absolute power during the first three or four time blocks and then growing the band power at the end of the session, Fig 8, especially in the left panel. For RSeC the highest absolute power can be observed for the first 4 days of BASE whereas during RSeO the largest amplitude of power band was during recovery period. These changes in brainwave power result in higher variance (visible in Fig 6) and thus it is advisable to test for differences on time-dependent powers. What we found intriguing, is the values of alpha power in both sleep restriction (SR) and recovery periods (RCV) being greater than in the control period in eyes open condition.

## Discussion

### Recovery process

The primary goal of the study was to investigate the recovery process following an extended period of sleep restriction. We have observed differences in behavioral, motor, and neurophysiological responses to both sleep loss and recovery. The main results are summarized in Table 1: full return (reaction times in behavioral task), only partial return (actigraphy, behavioral task accuracy), or no return (ERPs, power spectrum) to the baseline values within the seven days of recovery.

**Table 1:** A simplified summary of the results where the difference between baseline and sleep restriction or recovery was found significant. The arrows ↑ and ↓ indicate increase or decrease of a given measure. Partial return indicates a tendency for a given measure to go back to baseline value, even if they still remain different. * FCz amplitude at 272 ms, Pz at 600 ms. ** Delta, theta and alpha bands at Cz; delta and alpha bands at O1, O2.

	Quantity	BASE→SR	SR→RCV	Reference
Actigraphy	Rest CCDF exponent *γ* (ZCM,PIM, ZCM and/or PIM)	yes ↓	partial ↑	Fig 2G and 2H Fig 9 in S1 File left
	Activity CCDF parameter *α* (ZCM, ZCM or PIM)	yes ↑	partial ↓	Fig 9 in S1 File right
Behavioral	Accuracy	yes ↓	partial ↑	Table 2 in S1 File
	RTs	yes ↑	yes ↓	Fig 3
	RTs (median)	yes ↑	partial ↓	Sec. “Behavioral measures”
	After-pause RT boost	partial ↑	yes ↑	
EEG	FCz amplitude*	yes ↓	yes ↓	Fig 5 top
	Pz amplitude*	yes ↓	no	Fig 5 bottom
	ERP latency	yes ↓	yes ↓	Sec. “EEG data: ERP analysis”
	Cz band powers**	no ↓	yes ↓	Figs 6–8,
	O1, O2 and band powers**	yes ↓	no ↑	Table 4 in S1 File

The literature provides varied data as to the length of necessary recovery sleep after prolonged sleep restriction. Philip et al. [56] observed full recovery to baseline levels of performance and objective alertness after just eight hours of recovery sleep following 5-night sleep restriction to four hours (the same regarded one night of total sleep deprivation). An important issue was the age of the participants—this study comprised 18 healthy middle-aged subjects (46–55 years), so the conclusion may not be valid for younger individuals who typically need more sleep. It seems coherent with other observations confirming that aging is connected with better tolerance to sleep loss (e.g., [57, 58]), which is one of the very few advantages of older age, apart from ‘positivity’ in emotion regulation and increase in crystallized intelligence.

The studies involving younger participants showed that recovery from sleep restriction to four hours during 5–7 nights is not complete during subsequent resting period of 3 to 7 nights of normal sleep. The sleep-dose response study conducted by Belenky et al. [17] showed the reduced brain operational capacity persisting for three days of normal sleep duration and thought to be the effect of former adaptation to chronic sleep restriction [19]. Described the positive effects of three recovery nights on daytime sleepiness, fatigue and cortisol levels but not on performance compromised with six sleep restriction nights. Rupp et al. [23] reported the effects of 5-night recovery after 7-night restriction—the authors suggested that the rate at which recovery sleep reverses the alertness and performance impairments depends on the amount of sleep obtained before the restriction period. It means that the physiological mechanisms underlying chronic sleep debt should be considered as long-term adaptive changes. Axelsson et al. [21] concluded that seven days of recovery after five days of restricted sleep (to 4 hours a night) allowed participants to return to the baseline for sleepiness and median reaction time, but not for lapses.

Bougard and colleagues [20] looked at the consequences of a 7-day sleep restriction (to 4 hours) and the following 13-day recovery stage. They have looked at microsleeps during the day, wakefulness capacities, and feelings of sleepiness during the morning and evening time measurements. It has been found that the number of microsleeps, as measured by the EEG, has increased respectively to the homeostatic sleep pressure with the accumulation of the SR days. Interestingly, the number of microsleeps was still high after the first night of recovery and only returned to baseline levels after 12 days, which suggests that after a prolonged period of sleep restriction, participants’ recovery processes are slow and complex to adjust to, which are much slower than in the case of acute sleep restriction [17]. There were no differences in the observed number of microsleeps in the morning and evening sessions. In this study, all sleep-related measures returned to baseline on the 12th day of the recovery stage. The results obtained by Bougard et al. [20] appear consistent with one-week recovery, as in our experiment, not being sufficient to observe behavioral (accuracy) and neurophysiological parameters (EEG) returning to baseline levels after 10-day sleep restriction. The nature of the study itself imposes several limitations. Almost all the experiments referred to in this paper were conducted entirely in laboratory conditions; in one case, the participants spent a week of the 13-day recovery at home (wearing wrist activity monitors). Participants in our study were spending their nights (and most of the daytimes) in the so-called ‘natural conditions’; they were asked to follow their daily routines. Naturally, the price to pay for this paradigm was a number of uncontrollable factors that might have affected their lifestyle, alertness, and mood, which may cause potential caveats in the interpretation of results.

The multi-faceted character of the study allowed us to investigate the specificity of the recovery process. First, of behavioral measures, only mean RT reverts to baseline values, while some others only partly (accuracy, RTs median). Secondly, the actigraphy measures revert to normal only to an extent, which suggests long-lasting (even a week) disruption of motor control and overall behavior, including daily routines. Lastly, the EEG measures either reveal no significant change between conditions (power spectrum in the task and resting state with eyes closed at Cz) or, interestingly, the change from the baseline is not reverted by the recovery (ERPs and power spectrum).

The current study suggests that 7-day recovery following 10-day sleep restriction is sufficient only for the reaction speed to reverse to baseline, while the other behavioral, locomotor, and neurophysiological measures do not show such improvement. A great diversity of research procedures and proportions of sleep restriction and recovery times makes it hardly possible to compare our results with those reported in the literature. However, we note a few similar findings pointing to the incomplete restoration of some behavioral indices: Axelsson et al. [21] observed improvement in mean RT but not in the number of lapses, and Pejovic and colleagues [19] reported no improvement in RT parameters (median, lapses, fastest and slowest responses) even when objective sleepiness returned to baseline.

### Actigraphy

Many studies treat actigraphy simply as a tool for monitoring compliance with the sleep protocol at night. Yet, it is sensitive enough to measure both daily and nocturnal locomotor activity patterns reflecting altered psychomotor behavior associated with several psychiatric diseases and disorders [59]. Actigraphic measures follow universal distributions, but the universality may break down in disorders or conditions influencing brain functions [60–63]. Specifically, changes in locomotor activity can be an indicator of sleep deficiency [12] or a response to sleep deprivation treatment [13].

Relative to the whole body of literature on actigraphy in sleep research, few studies associate accelerometry-based quantities with self-reported, behavioral, or neural measures in humans. Noteworthy exceptions are a large cross-sectional study reporting associations between disrupted circadian rhythmicity of rest-activity and, among others, mood and reaction times [64] and a study on fatigued drivers combining actigraphy, behavioral data and EEG (power spectra and their causal relations between frontal and parietal midline brain areas) [65]. The same combination of actigraphy, behavioral and EEG measures used in our study proved to be a valuable approach to recovery process monitoring.

### EEG data

In terms of EEG measures, alpha band power was reported to be either positively correlated with sleepiness (or length of wakefulness) in task EEG [66] and resting state eyes open [54] or negatively with eyes closed [33, 67] (the last case reportedly explained by the increased amount of microsleep [54]), which agrees with our results. The outcomes for theta power at Cz and frontal electrodes do not replicate some established results: among the known sleep deprivation hallmarks is an increase in the theta range (4–8 Hz) power in wake EEG (open eyes) [54, 68], especially the frontal theta (closed eyes) [33], positively correlated with subjective sleepiness during wake task EEG [66] and with pre-existing sleep deprivation during sleep inertia period [69], and connected, e.g., to changes in body temperature [70]. The increased theta power, however, is only mentioned in the context of acute sleep deprivation (usually, up to 40 hours of wakefulness) [10] or a short sleep restriction (two days [71]), whose experimental designs are hardly comparable with ours. Moreover, participants in our study had their EEG measurements close to the time of their optimal wakeful functioning—quite the opposite to what happens during prolonged wakefulness in the studies cited above. Not being able to replicate the theta power increase might thus be a result of too small a sample size in our study or possible interactions with the duration and acuteness of sleep restriction and time of day.

Next, the ERP results beg the question if they might be affected by habituation to the task. The P300 amplitude—but not its latency –- has been shown to habituate after repeated presentation of visual stimuli [72, 73], as the task becomes more automatic and thus attentional processes might be reduced. After several days of practice also the P300 latency can be reduced, as evidenced [74] in a Go/No-go paradigm for No-go stimuli. At the same time P300 amplitude was shown to decrease and its latency to lengthen in response to sleep deprivation [75–77], however, these studies dealt with acute sleep deprivation and auditory task only.

On the other hand, our current result is consistent with Oginska et al. [78]. In that study, one group of subjects first went through one week sleep restriction and later, after a break, through a week of unrestricted sleep, while the second group had the order reversed. The reduction of P300 amplitude in the sleep restriction period with respect to unrestricted sleep was found irrespective of the experimental group, which rules out attributing the changes merely to habituation to repeated stimuli. Additionally, it is worth noting that the EEG resting state as well as actigraphy results are more agnostic to study design.

### Conclusion

Our results regarding the recovery process specificity, i.e. non-uniform return of different measures to baseline levels, could be interpreted from two perspectives—local sleep phenomenon or adaptive brain mechanisms. The idea of a so-called local sleep, meaning that only some brain regions might be asleep whilst other are not, has become more prominent in sleep studies [79]. Those off-periods have been initially found in animal studies, which showed that when sleep-deprived rats were awake, there were some local neurons that resembled the sleep-states in their activity [80]. Additionally, it has been found that such off-states are also linked with decreased performance and tend to spread and change from local to global under sleep deprivation and an increased need for sleep. This was associated with an increased theta band frequency and poorer performance in rats [80]. Other studies have previously shown that such an increase is typically seen in humans following a period of sleep deprivation, and performance errors [81] Since then, such periods of silence have been reported in adults [82, 83] and children [84]. The existence of such off-periods during wakefulness suggests that the previous, existing sharp division between “asleep” versus “awake” brain should be questioned. Therefore, the presence of the local sleep during wakefulness could account for poorer cognitive, behavioral and motor performance in individuals following a period of sleep restriction. Moreover, according to Hudson et al. [11]) the occurrence of local sleep may explain the link between the vigilance decrement and monotony, i.e. persistent use of the same brain circuitry. In our experiment, both sleep loss and monotony were key factors, so the local sleep interpretation seems to be justified.

The other explanation is that the neural activation patterns learned, and possibly optimized, during the demanding sleep conditions become consolidated. It is consistent with the suggestion given by Belenky et al. (2003), that “the brain undergoes adaptive changes in response to chronic sleep restriction, that serve to sustain a stable (albeit reduced), level of performance” [85]. The authors further suggest that “these changes persist into the recovery period and prevent a rapid return to baseline performance”. One may assume here some modifications of sleep architecture throughout prolonged sleep restriction (i.e. compensatory increases in slow-wave or REM sleep); other mechanisms, like changes in endocrine secretion, may also be taken into account.

In the outlook, it might be worthwhile to disentangle the early and late recovery effects (the first one-two days versus a week or more)—our current sample size, however, is insufficient for robust hypothesis testing of daily measurements—and to extend the recovery period at least for the sake of actigraphy recording.

We are also still aiming to look at the correlations between performance and various (reported) neurophysiological quantities, especially in the light of the recently reported decline of long-range temporal correlations in the human brain during sustained wakefulness [86, 87] and the correlation of neural and behavioral scaling laws [88]. Another topic not explored here is the topographic distribution of the changes in neural activity, which are known to differ for different tasks after total sleep deprivation [71, 89].

### Limitations

To assess and factor out the inter- and intra-individual variability—which are known to be considerable, e.g., for alpha rhythms [52, 53]—would also involve a considerably larger, possibly a multi-center study. As mentioned above, this exploratory study was conducted in natural conditions that may induce some other problems. For example, participants’ caffeine intake was not controlled. The subjects were instructed to refrain from caffeine consumption before visiting the laboratory but due to the nature of the study and the extended period of sleep deprivation, they reported that they might not be able to meet the expectations of the study and restrain themselves from napping. Therefore, we made a decision that caffeine consumption was allowed in order for the participants to finish the study according to the prescribed schedule, but the quantities of caffeine drinks were not recorded. Besides, we have learned that caffeine appears to have limited efficacy for maintaining alertness during prolonged sleep restriction. However, there may be some costs associated with its use for recovery from sleep loss ([90]).

After all, some participants were not able to comply strictly with their reduced sleep schedules—such cases were monitored and revealed by actigraphy, but resulted in drop-outs. Because of that and the costs, the sample size is relatively small for an EEG study. There was also no control group, where a subject would attend 21 consecutive days of EEG measurements without any sleep restrictions. Due to the mere length and tediousness of the study one has to take into account, e.g., that in Stroop task the return to baseline accuracy might be only partial, because of the participants’ decrease in motivation. This as well invites questions about habituation effects, which have been, however, addressed before. Finally, there are stable, trait-like inter-individual differences in the vulnerability to sleep deprivation [91–93], which are not controlled in our study. On the other hand, we would like to emphasize how demanding—owing to its extended nature, subjects’ non-compliance, their increased fidgetiness having an EEG cap on, falling asleep in resting state with eyes closed, etc.—is acquisition of quality data on chronic sleep restriction.

We are aware of the discrepancies between subjective assessments and objective measures of performance and neural functioning which make problematic the descriptions of individual vulnerability to sleep loss or monitoring the recovery processes after sleep deprivation. In this study we observed the differences between some objective measures—performance variables (RT) and neurophysiological indices (ERPs and power spectrum). Which one should be the criterion of the normal functioning? In our opinion, the recovery process is complete only when all the measures return to the baseline levels.

Prolonged periods of sleep restriction seem to be common in contemporary ‘regular day workers’, including students. Although the saying “Sleep is for the weak” is no more the mantra of workaholics and ambitious individuals, we all treat restricted sleep as a norm. Some studies, as the one reported above, convince us that neurobehavioural consequences of chronic partial sleep deprivation cannot be overcome easily and last much longer than one expects.

## Supporting information

S1 File (PDF)
