# Supplementary material for: Observing changes in human functioning during induced sleep deficiency and recovery periods
Source: PLoS One. 2021 Sep 1;16(9):e0255771. doi: 10.1371/journal.pone.0255771 (PMC8409667; doi:10.1371/journal.pone.0255771)
Supplement: S1 File — (PDF) [file pone.0255771.s001.pdf]

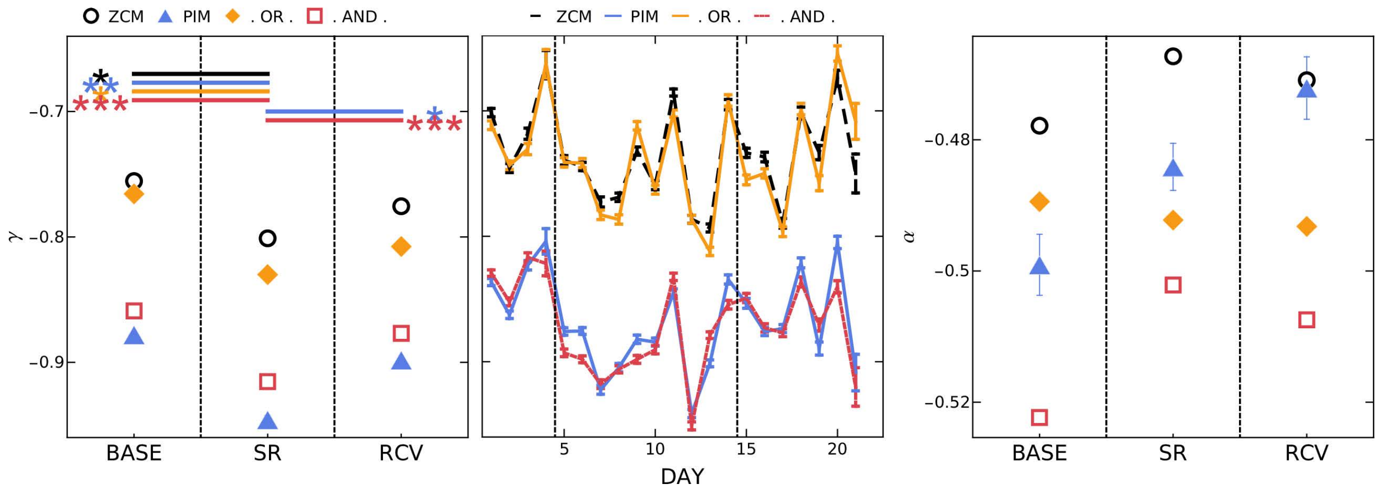

Fig 9. (Left panel) The gamma exponent of cumulative distribution $C(a)$ of rest periods (defined as below ZCM, PIM, ZCM **or** PIM threshold, ZCM **and** PIM threshold) in the 3 measured conditions: baseline, sleep restriction and recovery, and (middle panel) as function of consecutive days. Asterisks denote significance of differences between conditions, color coded for the respective rest thresholds. The exact test results for PIM are given in Sec. Results: Actigraphy. (Right panel) The alpha exponent of cumulative distribution $C(a)$ of activity state (defined as above respective thresholds)
.

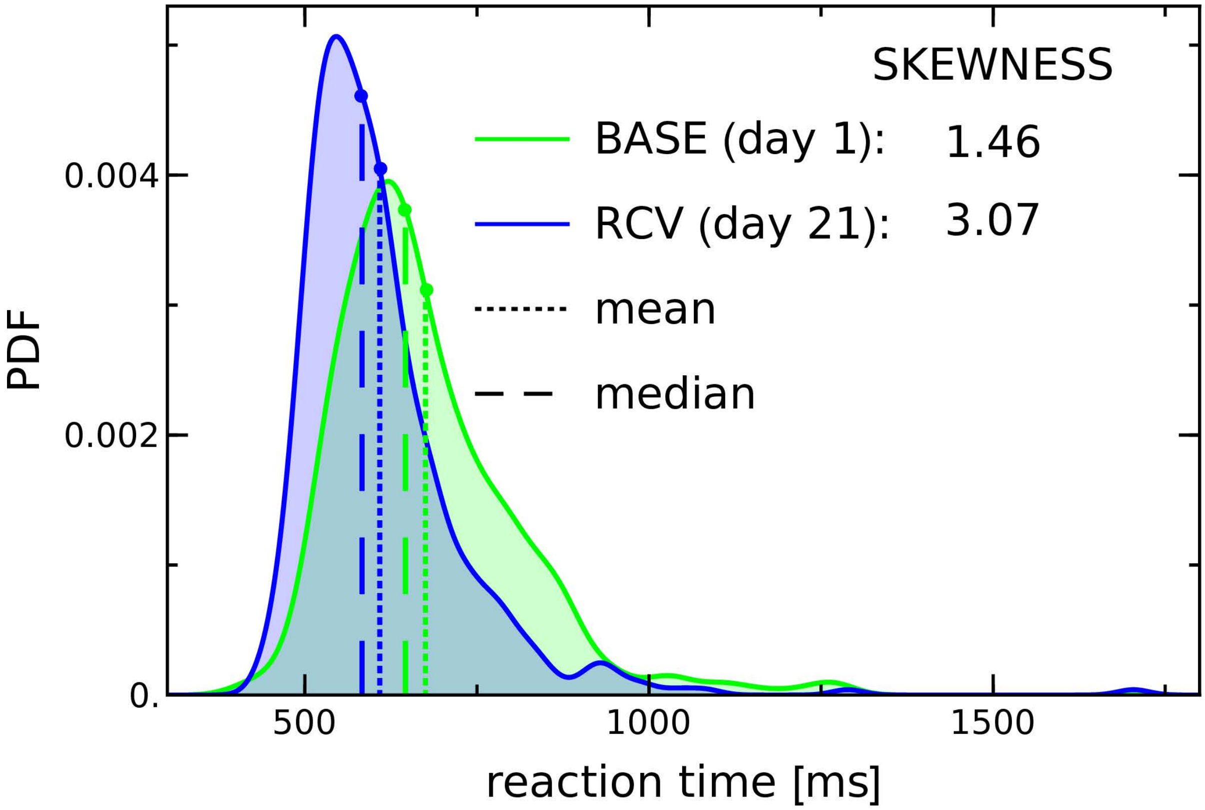

Fig 10. Histogram of reaction times for a single subject on the first and last day of the experiment. The distributions are highly skewed to the right, which results in significant differences between the mean and median.

Table 2. Average changes in accuracy and reaction times between the beginning and end of an experimental session (432 stimuli, 22 minutes) computed from linear regression slopes, and the drop from linear fit to RTs shortly after pauses in between session blocks. Beside means (or medians) and 95% CI for each condition on the left, we also report on p-values for pairwise differences between conditions (e.g., the element in the 1st row and 2nd column gives the p-value for BASE-SR). These results are summarized in Sec. Behavioral measures.

ACCURACY

			p-values		
	mean [%]	95% CI	BASE	SR	RCV
BASE	-0.072	[-0.66, 0.80]	–	$\mathbf{1.1 \times 10^{-5}}$	**0.00051**
SR	-1.96	[-2.55, -1.37]		–	0.19
RCV	-1.57	[-2.22, -0.91]			–

REACTION TIMES

			p-values		
	mean [ms]	95% CI	BASE	SR	RCV
BASE	24.3	[18.7, 29.9]	–	**0.00019**	0.24
SR	37.5	[32.6, 42.4]		–	$\mathbf{4.9 \times 10^{-6}}$
RCV	21.6	[16.4, 26.8]			–

REACTION TIMES (medians)

			p-values		
	median [ms]	95% CI	BASE	SR	RCV
BASE	17.9	[12.2, 23.7]	–	**0.049**	0.22
SR	23.7	[19.8, 27.6]		–	0.16
RCV	20.8	[16.2, 25.3]			–

AFTER-PAUSE RT DROP

			p-values		
	mean [ms]	95% CI	BASE	SR	RCV
BASE	-20.8	[-30.2, -11.3]	–	**0.0045**	$\mathbf{1.2 \times 10^{-5}}$
SR	-21.8	[-30.1, -13.4]		–	0.09
RCV	-37.6	[-46.4, -28.8]			–

Table 3. P-values of Kruskal-Wallis test with BASE, SR, RCV conditions as described in Methods: EEG section and Results: EEG data: power spectrum analysis.

DIFFERENCES IN EEG POWER

		delta	theta	alpha	beta
Cz	RSeO	**0.010**	**0.041**	0.83	**0.017**
	RSeC	0.96	1	0.08	0.94
F3	RSeO	0.53	0.8	0.6	0.33
	RSeC	0.47	0.48	0.12	0.60
F4	RSeO	0.51	0.54	0.41	0.75
	RSeC	0.69	0.66	0.051	0.32
O1	RSeO	**0.04**	0.6	0.82	0.59
	RSeC	0.42	0.39	**0.012**	0.12
O2	RSeO	**0.05**	0.36	0.69	0.87
	RSeC	0.53	0.66	**0.044**	0.57

Table 4. P-values of Conover-Iman post hoc test following Tab. 3.

		BASE-SR	BASE-RCV	SR-RCV
Cz	RSeO, delta	0.14	**0.0068**	0.22
	RSeO, theta	0.62	**0.046**	0.13
	RSeO, beta	0.36	**0.015**	0.12
O1	RSeO, delta	**0.03**	0.26	0.58
	RSeC, alpha	**0.0084**	0.084	0.65
O2	RSeO, delta	0.075	0.82	0.17
	RSeC, alpha	**0.033**	0.17	0.74
